# Safety and immunogenicity of the PRAME cancer immunotherapeutic in metastatic melanoma: results of a phase I dose escalation study

**DOI:** 10.1136/esmoopen-2016-000068

**Published:** 2016-08-08

**Authors:** R Gutzmer, L Rivoltini, E Levchenko, A Testori, J Utikal, P A Ascierto, L Demidov, J J Grob, R Ridolfi, D Schadendorf, P Queirolo, A Santoro, C Loquai, B Dreno, A Hauschild, E Schultz, T P Lesimple, N Vanhoutte, B Salaun, M Gillet, S Jarnjak, P M De Sousa Alves, J Louahed, V G Brichard, F F Lehmann

**Affiliations:** 1 Skin Cancer Center Hannover, Hannover Medical School, Hannover, Germany; 2 Unit of Immunotherapy of Human Tumors, Fondazione RCCS Istituto Nazionale dei Tumori, Milan, Italy; 3 Petrov Research Institute of Oncology, St. Petersburg, Russia; 4 Istituto Europeo Di Oncologia, Milano, Italy; 5 Skin Cancer Unit, German Cancer Research Center (DKFZ), Heidelberg, Germany; 6 Department of Dermatology, Venereology and Allergology, University Medical Center Mannheim, Ruprecht-Karl University of Heidelberg, Mannheim, Germany; 7 Melanoma, Cancer Immunotherapy and Innovative Therapies Unit, Istituto Nazionale Tumori Fondazione Pascale, Naples, Italy; 8 Cancer Research Center, Moscow, Russia; 9 Aix Marseille University Hôpital de la Timone, Service de Dermatologie, Marseille, France; 10 Immunotherapy Unit, Romagna Cancer Institute IRST- IRCCS, Meldola, Italy; 11 Department of Dermatology, University Hospital Essen, Essen, Germany; 12 Istituto Nazionale per la Ricerca sul Cancro Oncologia Medica, Genova, Italy; 13 Humanitas Cancer Center, Istituto Clinico Humanitas IRCCS, Rozzano, Italy; 14 Department of Dermatology, University of Mainz, Mainz, Germany; 15 Dermatology Clinic, Hôpital Hôtel-Dieu, CHU Nantes, Nantes, France; 16 Department of Dermatology, Skin Cancer Center, Schleswig-Holstein University Hospital, Kiel, Germany; 17 Department of Dermatology, Paracelsus Medical University, Klinikum Nuremberg, Nuremberg, Germany; 18 Département d'Oncologie Médicale, Centre Eugène Marquis, Rennes, France; 19 GSK Vaccines, Rixensart, Belgium; 20 Celyad, Mont-Saint-Guibert, Rixensart, Belgium; 21 Vianova-Biosciences, Lasne, Belgium

**Keywords:** metastatic melanoma, PRAME antigen, safety, immunogenicity, cancer immunotherapy

## Abstract

**Purpose:**

The PRAME tumour antigen is expressed in several tumour types but in few normal adult tissues. A dose-escalation phase I/II study (NCT01149343) assessed the safety, immunogenicity and clinical activity of the PRAME immunotherapeutic (recombinant PRAME protein (recPRAME) with the AS15 immunostimulant) in patients with advanced melanoma. Here, we report the phase I dose-escalation study segment.

**Patients and methods:**

Patients with stage IV PRAME-positive melanoma were enrolled to 3 consecutive cohorts to receive up to 24 intramuscular injections of the PRAME immunotherapeutic. The RecPRAME dose was 20, 100 or 500 µg in cohorts 1, 2 and 3, respectively, with a fixed dose of AS15. Adverse events (AEs), including predefined dose-limiting toxicity (DLT) and the anti-PRAME humoral response (ELISA), were coprimary end points. Cellular immune responses were evaluated using in vitro assays.

**Results:**

66 patients were treated (20, 24 and 22 in the respective cohorts). AEs considered by the investigator to be causally related were mostly grade 1 or 2 injection site symptoms, fatigue, chills, fever and headache. Two DLTs (grade 3 brain oedema and proteinuria) were recorded in two patients in two cohorts (cohorts 2 and 3). All patients had detectable anti-PRAME antibodies after four immunisations. Percentages of patients with predefined PRAME-specific-CD4+T-cell responses after four immunisations were similar in each cohort. No CD8+ T-cell responses were detected.

**Conclusions:**

The PRAME immunotherapeutic had an acceptable safety profile and induced similar anti-PRAME-specific humoral and cellular immune responses in all cohorts. As per protocol, the phase II study segment was initiated to further evaluate the 500 µg PRAME immunotherapeutic dose.

**Trial registration number:**

NCT01149343, Results.

**Key questions**

**What is already known about this subject?**

- Checkpoint-inhibitors are successfully used for the treatment of metastatic melanoma, but more specific, tumour antigen-targeted immunotherapies would be desirable to increase specificity and decrease side effects of immunotherapeutic approaches.
- The human tumour antigen PReferentially expressed Antigen of MElanoma (PRAME) is a potential candidate because it is expressed by several tumour types, including melanoma. T-cell immune responses have been induced in previous studies, with no safety issues raised.

**What does this study add?**

- This phase I/II dose-escalation study evaluated the PRAME cancer immunotherapeutic (three dosages of recPRAME+AS15 immunostimulant) in patients with advanced malignant melanoma.
- Two weeks post-treatment 4, the safety profile was clinically acceptable at all three dosages investigated.
- Treatment-induced robust humoral immune responses in all patients.
- Specific PRAME-reactive CD4+ T-cells responses were observed at all three dosages, although CD8+ T-cell immunogenicity was barely detectable and CD8+ T-cells responses were absent.
- As per protocol, the highest dose was selected for assessment of clinical activity in the phase II segment of the study.

**How might this impact on clinical practice?**

- This study provides evidence that PRAME is a possible target for specific immunotherapy in melanoma. Initial implication for clinical practice will be assessed in the phase II study segment.

## Introduction

Cutaneous melanoma is the most aggressive form of skin cancer and patients with metastatic disease have a poor prognosis.^1^ The therapeutic landscape of advanced melanoma changed significantly since 2011 with the availability of checkpoint inhibitors (ipilimumab, nivolumab and pembrolizumab), selective inhibitors of V600-mutated BRAF (vemurafenib, dabrafenib and their combination with MEK-inhibitors cobimetinib and trametinib, respectively) as well as further investigation of antibodies against PD-L1.^2^ Anticheckpoint therapies are associated with potentially serious adverse events (SAEs), notably autoimmune-related toxicity, and few patients derive long-term benefit.^2^ Furthermore, development of resistance (BRAF inhibitors) also occurs frequently.

In the context of these advances, the aim of specific immunotherapies is to induce clinically significant, long-lasting responses with moderate toxicity. The human tumour antigen PReferentially expressed Antigen of MElanoma (PRAME) was originally identified as the target antigen of a cytolytic T-lymphocyte clone derived from a patient with melanoma.^3^ *PRAME* is expressed in low levels in a normal ovary, endometrium, kidney and adrenal tissues,^3^ and overexpressed in a range of cancers including 95% of metastatic melanoma tumours.^3^ PRAME expression is associated with an unfavourable prognosis in some solid tumours including breast cancer.^4^

PRAME is a potential candidate for cancer immunotherapy because it is expressed by a variety of tumours and can induce T-cell immune responses.^3^ ^5–8^ In a phase I study, a combined plasmid-peptide vaccine derived from PRAME and prostate-specific membrane antigen was administered to patients with metastatic solid tumours who had failed standard treatment options.^9^ Expansion of PRAME-specific T-cells was observed and no safety issues were identified.

In a dose-escalation phase I study, we sought to determine an adequate dose of a recombinant PRAME protein (recPRAME, GSK, Belgium) administered with GSK's proprietary immunostimulant AS15, through evaluation of the safety and immunogenicity of the PRAME immunotherapeutic in patients with PRAME-positive metastatic melanoma. Here we present safety and immunogenicity data two weeks after dose 4 that led to dose selection according to protocol-defined rules. A phase II study segment is ongoing and will assess clinical activity of the selected dose of recPRAME. Clinical activity observed in phase I will be described at the time of the final analysis.

## Methods

The open-label, phase I dose-escalation study (http://www.clinical trials.gov NCT01149343) study protocol was approved by institutional review boards at each participating centre. Written informed consent was obtained from each patient prior to the performance of any study-specific procedures, including PRAME screening.

Overall, this study was conducted in accordance with the principles of ‘good clinical practice’, the principles of the Declaration of Helsinki and all applicable regulatory requirements. During the course of the study, whenever potential or actual issues with regard to the conduct of the study were identified, either via site monitoring activities or brought to GSK's attention by other oversight mechanisms, these issues were investigated and, where possible, appropriate corrective and/or preventive actions were taken.

Coprimary objectives were to document and characterise, for each dose of the PRAME immunotherapeutic, the dose-limiting toxicities (DLTs) and the anti-PRAME humoral immune response. Secondary objectives included evaluation of additional indicators of safety and immunogenicity in terms of antigen-specific cell-mediated immune (CMI) responses.

### Patients

Patients were ≥18 years of age with histologically proven cutaneous PRAME antigen-positive melanoma. Eligible patients had stage IV M1b-c melanoma, including completely resected stage IV patients except those with IV M1c disease with serum lactate dehydrogenase >1.5 times the upper limit of normal, or with active involvement of the central nervous system. See the supplemental data for details on inclusion/exclusion criteria.

10.1136/esmoopen-2016-000068.supp1 supplementary data

### Treatment regimen

The PRAME immunotherapeutic (recPRAME+AS15) was administered intramuscularly into the deltoid or thigh. The composition of the PRAME immunotherapeutic is provided in the online supplementary data.

Escalating doses of recPRAME (20, 100 or 500 µg) combined with a fixed dose of AS15 were evaluated in three consecutive cohorts. A maximum of 24 doses of PRAME immunotherapeutic could be administered. The treatment schedule is provided in the online supplementary data. Enrolment was staggered to allow early identification of safety signals, and protocol-defined rules determined when dose escalation to the next level could occur (see online supplementary data).

### Assessment of safety

A DLT was defined as any of the following AEs considered related or possibly related to administration of the PRAME immunotherapeutic: (1) ≥grade 3 AE (grade 3 myalgia, arthralgia, headache, fever, rigors/chills and fatigue were to have persisted for 48 hours despite therapy in order to be considered as a DLT); (2) ≥grade 2 allergic reaction occurring within 24 hours postinjection of the PRAME immunotherapeutic; (3) any decrease in renal function with a creatinine clearance <40 mL/min considered related or possibly related to the PRAME immunotherapeutic; or (4) any symptomatic and confirmed adrenal insufficiency related or possibly related to the PRAME immunotherapeutic.

### Immunogenicity

#### Humoral immunity

Anti-PRAME IgG antibodies were measured by ELISA prior to administration of dose 1, 2 weeks postdose 2 and 2 weeks postdose 4, as described in the online supplementary data. A response was defined as postimmunisation anti-PRAME antibody concentration ≥12 EU/mL (defined from 102 healthy donors) in initially seronegative patients (seroconversion); and an increase of ≥2fold in initially seropositive patients.

#### Cell-mediated immunity

CMI was measured prior to the first dose and 2 weeks postdose 4 as described in the online supplementary data. PRAME T-cell immunogenicity (characterised by detection and quantification of T-cells producing both interferon-γ (INF-γ) and tumour necrosis factor α (TNF-α) in an in vitro assay) cut-off scores for a positive response were defined from a panel of healthy donors (n=23, cut-off 2.68 for CD4+ T-cell analysis and 1.15 for CD8+ T-cell analysis). A patient was considered as a T-cell responder (CD4+ or CD8+) if the ratio of immunogenicity scores between a positive postimmunization sample and its corresponding baseline was ≥4 fold.

### Dose selection criteria

The dose was selected based on safety and immunogenicity data. A dose was considered adequate if ≤two cases of DLT were reported at any time among the 15 patients in each cohort; and if the dose showed ≥70% (≥11/15) anti-PRAME antibody responses after four immunisations. If more than one dose level satisfied safety and humoral immune response criteria, the selection of the dose would also take into account CMI responses. If the best immunological dose could not be determined by applying these criteria, the highest dose with acceptable safety and immunogenicity was selected.

### Statistical analysis

The study was descriptive and no comparative tests were performed. See the online supplementary data for definitions of the total treated cohort and the according-to-protocol (ATP) cohort for immunogenicity, and for information on statistical programs used.

## Results

A total of 138 patients were screened for *PRAME* expression and 66 were enrolled (see online supplementary figure S1). Most patients (97.0%) were Caucasian and the mean age was 60.2 years. There were more stage IV M1b patients in cohort 2 than in the other cohorts (table 1).

**Table 1:** Demographic and disease characteristics (total treated cohort)

Characteristic	Cohort 1 (20 μg) N=20	Cohort 2 (100 μg) N=24	Cohort 3 (500 μg) N=22
Age at screening (years)			
Mean (SD)	60.3 (14.87)	60.8 (15.53)	59.5 (15.18)
Median	62.0	65.5	61.5
Range	22–81	27–84	20–81
Gender			
Female	7	11	10
Male	13	13	12
Disease stage			
IVM1b	4	12	5
IVM1c	10	11	12
IV NED	6	1	5
Prior therapies			
Interferon	5	7	6
Cancer vaccines*	3	3	5
Radiotherapy	5	3	0
Interferon+cancer vaccine*	0	1	2
Interferon+radiotherapy	2	3	0
Cancer vaccine*+radiotherapy	2	0	0
ECOG status			
0	16	18	22
1	4	6	0

*Cancer vaccines not containing PRAME antigen.

ECOG, Eastern Co-operative Oncology Group performance status; N, total number of patients; NED, no evidence of disease.

Across the three cohorts (20, 24 and 22 patients in cohorts 1, 2 and 3, respectively), 66 patients had received a total of 468 doses of the PRAME immunotherapeutic by the data lock point (DLP). The main reason for treatment discontinuation was disease progression (49/55 withdrawals). Two patients withdrew due to AEs not considered related to treatment: one patient in cohort 1 developed atrial fibrillation 5 days postdose 2 and one patient in cohort 2 developed dehydration on the day of dose 4. Two patients withdrew consent (not due to an AE) during treatment cycle 2. Until the DLP, two patients died from melanoma progression during treatment.

DLTs were reported by two patients. All were categorised as treatment-related grade 3 AEs. A patient in cohort 2 with small pre-existing residual local brain oedema after surgery and cerebral radiotherapy for brain metastases developed worsening of the focal brain oedema (ie, an about 5 mm increase) 5 days postdose 6. The patient recovered and continues the study treatment and remains disease-free. One patient in cohort 3 had microalbuminuria on the day of dose 8 and proteinuria 22 days postdose 8 (considered manifestations of a renal disorder). The microalbuminuria resolved and the patient was withdrawn due to progression of melanoma.

There were nine SAEs in six patients, including the DLT case of focal brain oedema, of which eight were considered as treatment unrelated. One potential immune-mediated disease was reported in a patient who developed grade 1 vitiligo on the day of dose 3, considered to be treatment-related. This patient demonstrated a humoral response but did not have a clinical response to treatment, nor CD4+ or CD8+ responses.

The majority of AEs reported from dose 1 were grade 1 or 2. There were 17 grade 3 AEs reported by 11 patients distributed across all three study cohorts and encompassing a wide range of conditions (see online supplementary table S1). No grade 4 AEs were reported. The most frequently reported AEs considered to be possibly related to immunisation were local symptoms at the injection site, influenza-like illness fatigue, chills, fever and headache (table 2).

**Table 2:** Summary of treatment-related adverse events* reported by at least two patients in any group (any grade)† from dose 1 until the data lock point, by maximum grade (total treated cohort)

	Cohort 1 N=20			Cohort 2 N=24			Cohort 3 N=22		
	Grade 1	Grade 2	Grade 3	Grade 1	Grade 2	Grade 3	Grade 1	Grade 2	Grade 3
Adverse event	n	n	n	n	n	n	n	n	n
Not yet coded	1	0	0	1	0	0	1	0	0
Injection site reaction	6	4	0	10	3	0	8	5	0
Fever	3	2	0	5	1	0	5	1	0
Influenza-like illness	2	2	0	3	1	0	6	0	0
Fatigue	2	1	0	2	0	0	2	3	0
Headache	4	1	0	0	2	0	1	2	0
Chills	4	0	0	1	0	0	2	2	0
Asthenia	1	0	0	0	0	0	2	4	0
Myalgia	4	0	0	1	1	0	0	1	0
Nausea	5	0	0	1	0	0	0	1	0
Arthralgia	0	1	0	1	1	0	0	0	0
Bone pain	1	1	0	0	0	0	0	0	0

*AEs were assessed according to the Common Terminology Criteria for Adverse Events V.4.0, and coded to the preferred term level using the Medical Dictionary for Regulatory Activities.

†See online supplementary table S1 for all treatment-related adverse events from dose 1 until the data lock point, by maximum grade (total treated cohort).

N=number of patients with at least one administered dose; n=number of patients reporting the adverse event at least once.

In addition to the case of DLT, proteinuria also occurred in one patient in cohort 1 (grade 1: onset on the day of dose 3 and lasting 15 days) and one patient in cohort 2 (maximum severity grade 2) on two occasions (onset 14 days postdose 3 lasting 10 days and onset on the day of dose 9, ongoing at the DLP). These events were considered unrelated to the PRAME immunotherapeutic.

There were no grade 4 laboratory abnormalities. Grade 3 laboratory abnormalities occurred in seven patients (3 in cohort 1, 1 in cohort 2 and 3 in cohort 3) and included anaemia (2 patients), increased γ-glutamyl transpeptidase (3 patients, plus one patient with concomitant increased alkaline phosphatase), increased lymphocyte count (1 patient).

One patient (cohort 3) was reported to have grade 1 adrenal insufficiency concomitantly with decreased blood cortisol levels at dose 5. This finding was not reported as an AE.

**Figure 1:**
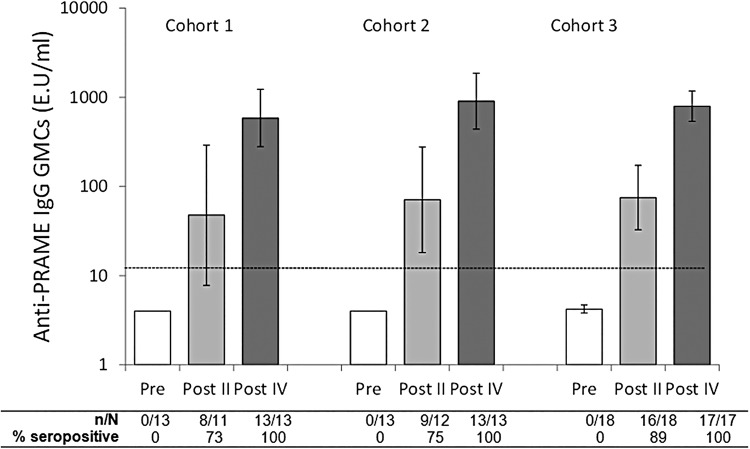
Seropositivity rates and geometric mean antibody concentrations (GMCs) for anti-PRAME IgG antibodies (ATP cohort for immunogenicity). Footnote: N=number of patients with available results, n/%=number/percentage of patients with concentrations above the cut-off, vertical lines indicate 95% CIs, dotted line shows assay cut-off (12 E.U/mL), Pre=prior to dose 1, Post II=2 weeks after the second dose, Post IV=2 weeks after the fourth dose. ATP, according-to-protocol; PRAME, PReferentially expressed Antigen of Melanoma.

All patients were seronegative for anti-PRAME IgG antibodies at baseline. At least 73% of patients in each cohort were seropositive after two doses. All patients had a humoral response (seroconversion) after 4 doses. Anti-PRAME antibody concentrations were higher after dose 4 than after dose 2 in all cohorts (figure 1).

**Figure 2:**
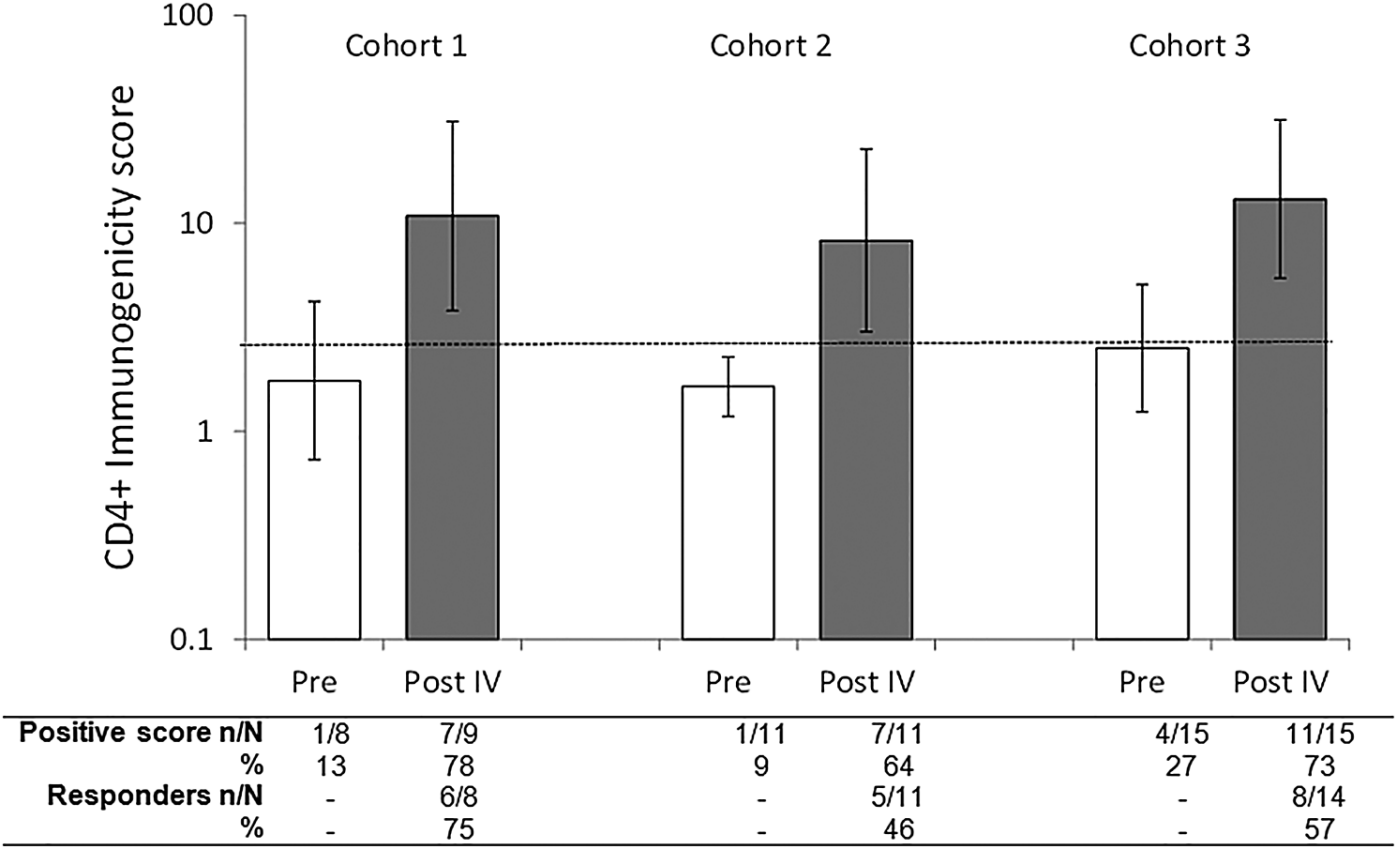
PRAME-specific CD4+ T-cell (TNF-α+/IFN-γ+) immunogenicity scores and cellular response prior to treatment and postdose 4 (ATP cohort for immunogenicity). Footnote: N=number of patients with available results, n/%=number/percentage of patients with immunogenicity score/response, vertical lines indicate 95% CIs, dotted line shows cut-off (2.68), Pre=prior to dose 1, Post IV=2 weeks after the fourth dose. See online supplementary data for details of the derivation of cut-offs and methods. ATP, according-to-protocol; IFN-γ, interferon-γ; TNF-α, tumour necrosis factor α; PRAME, PReferentially expressed Antigen of Melanoma.

After receiving dose 4, the number of patients with PRAME-specific CD4+ T-cell (TNF-α+/IFN-γ+) immunogenicity scores ≥cut-off was 7/9 in cohort 1, 7/11 in cohort 2 and 11/15 in cohort 3 (figure 2). No patients had CD8+ T-cell (TNF-α+/IFN-γ+) immunogenicity scores ≥cut-off before or after immunisation. Taking baseline immunogenicity scores into account, after four doses, the percentage of patients with PRAME-specific CD4+ T-cell response was 76%, 46% and 57% in cohorts 1, 2 and 3, respectively (figure 2). No patients presented CD8+ T-cell responses in any of the cohorts.

Out of all doses fulfilling the predefined criteria for dose selection, the 500 µg dose was selected for the phase II segment of the study as per protocol.

## Discussion

This dose-escalation study evaluated the PRAME immunotherapeutic in patients with advanced malignant melanoma. We observed *PRAME* expression in 84.6% of patients, which is in the range reported in the literature for metastatic melanoma.^3^

Antigen-specific cancer immunotherapeutics typically induce less toxicity than cytotoxic agents and non-specific immunotherapeutic treatments targeting T-cell checkpoints.^10^ ^11^ We conservatively predefined DLT based on the pattern of PRAME expression in healthy tissue, notably on potential adrenal and renal AEs. The PRAME immunotherapeutic had a clinically acceptable safety profile at all 3 doses investigated, consistent with another phase I study conducted in patients with non-small-cell lung cancer (NSCLC) in which no DLT was observed.^12^ The results of both studies, together with results of studies of the MAGE-A3 immunotherapeutic,^13–15^ support the acceptable safety profile of antigen-specific cancer immunotherapies.

No patients had pre-existing anti-PRAME antibodies and the PRAME immunotherapeutic induced a humoral immune response in all patients. Spontaneous anti-PRAME antibodies were reported in a small proportion of patients with NSCLC expressing PRAME, as was also the case for NSCLC and melanoma expressing MAGE-A3.^15^ ^16^ In contrast, baseline antibodies to other tumour antigens such as NY-ESO-1 are detected more frequently (eg, 16% for NY-ESO-1 in patients with melanoma^17^).

As observed in the parallel study in NSCLC (adjuvant setting), CD8+ T-cell immunogenicity was barely detectable or undetectable and CD8+ T-cells responses were absent. These results in patients with solid tumours contrast with studies in other clinical settings (patients with leukaemia where contact with circulating T-cells occurs) in which CD8+ T-cell responses to PRAME have been observed.^8^ CD8+ T-cells responses have been rarely described following active immunotherapy with recombinant proteins.^18^ Indeed, tumour-associated antigens present very low levels of CD8+ antigen-specific circulating precursor cells.^19^ Consequently, T-cell monitoring read-outs and their sensitivity and specificity have a direct impact on detectability of such weak T-cell responses. In contrast, PRAME-reactive CD4+ T-cell responses were observed in the three cohorts. CD4+ cells play a pivotal role in promoting CD8+ effector functions and in facilitating direct killing of tumour cells.^20^ ^21^ The combined actions of CD4+ cells and cytokines (INF-γ and TNF) induce tumour senescence.^22^ Thus, the presence of CD4+ T-cell responses in our study might be sufficient for an effective antitumour immune response mediated by the PRAME immunotherapeutic. The absence of detectable CD8+ T-cell immune responses does not exclude a clinical response. Work is continuing to identify means to improve CD8+ responses.

As observed with *MAGE-A3* expression in patients with melanoma,^23^ potential PRAME immunotherapeutic treatment-induced epitopic spreading as well as development of heterogeneity in tumour gene expression may affect treatment response. In eight patients with tumour progression during the study for whom an additional tumour sample was received, all eight remained PRAME-positive (data not shown). The persistence of the *PRAME* antigen expression into all progression/relapse lesions evaluated illustrates that a specific antigen loss variant does not seem to be the main mechanism of absence of clinical response to the PRAME immunotherapeutic.

In this phase I dose-escalation study, the PRAME immunotherapeutic 500 μg dose showed a clinically acceptable safety profile and was immunogenic, with humoral and specific CD4+ responses observed in the majority of patients. The highest dose was selected for assessment of clinical activity in the phase II segment of the study in melanoma.
