# Supplementary material for: Safety and immunogenicity of the PRAME cancer immunotherapeutic in metastatic melanoma: results of a phase I dose escalation study
Source: ESMO Open. 2016 Aug 8;1(4):e000068. doi: 10.1136/esmoopen-2016-000068 (PMC5070281; doi:10.1136/esmoopen-2016-000068)
Supplement: supplementary data [file esmoopen-2016-000068supp.pdf]

Safety and immunogenicity of the PRAME cancer immunotherapeutic in metastatic melanoma: results of a phase I dose escalation study

R. Gutzmer, L. Rivoltini, E. Levchenko, A. Testori, J. Utikal, P. A. Ascierto, L. Demidov, JJ. Grob, R. Ridolfi, D. Schadendorf, P. Queirolo, A. Santoro, C. Loquai, B. Dreno, A. Hauschild, E. Schultz, T. P. Lesimple, N. Vanhoutte, B. Salaun, M. Gillet, S. Jarnjak, P. M. De Sousa Alves, J. Louahed, V. G. Brichard, F. F. Lehmann

Supplementary data

Methods

The open-label, Phase I dose-escalation study (www.clinical trials.gov NCT01149343) was conducted in by 29 investigators in 6 countries (Czech Republic, Germany, France, Italy, Poland and the Russian Federation).

Inclusion criteria

Patients were to have an Eastern Cooperative Oncology Group (ECOG) performance status of 0 or 1, and adequate bone marrow reserve and renal, adrenal and hepatic function as determined using standard laboratory criteria.

Patients were to have PRAME antigen-positive tumor as determined by reverse transcriptase polymerase chain reaction (RT-PCR) analysis on fresh tissue samples [1].

Exclusion criteria

Patients were excluded if they had previously received systemic therapy for the metastatic disease. Previous adjuvant treatment with interferon, anti-CTLA-4

monoclonal antibody or a cancer vaccine containing a tumor antigen other than PRAME (such as the MAGE-A3 immunotherapeutic received as part of study www.clinicaltrials.gov NCT00796445) was allowed if the last dose was given eight weeks before the first dose of the PRAME immunotherapeutic. Isolated limb perfusion was allowed if performed at least four weeks before enrolment.

Patients requiring concomitant treatment with systemic corticosteroids or any other immunosuppressive agents were excluded (a maximum to 10 mg/day prednisone, or equivalent was permitted). Patients with previous or concomitant malignancies at other sites were excluded, as were patients with confirmed adrenal dysfunction, an autoimmune disease, patients who were known to be human immunodeficiency virus positive, patients with a family history of congenital or hereditary immunodeficiency or those with an uncontrolled bleeding disorder. Pregnant or lactating patients were not permitted to participate.

Treatment regimen

The recPRAME antigen is a PRAME-based recombinant protein of 626 amino acids (aa) produced in *Escherichia coli*. recPRAME was built as a fusion protein comprising the following structural elements: an N-terminal tripeptide containing the translator initiator methionine (M) and two unrelated aa (DP, aa 1-3), aa residues 20-127 of Protein D of *Haemophilus influenzae* (aa 4-111), the full-length 509-aa-long PRAME aa sequence (aa 112-620), a hexahistidine tag (aa 621-626) enabling the purification of the fusion protein by ion metal (Ni2+) affinity chromatography (IMAC) procedures. The immunostimulant AS15, is a combination of 3-O-desacyl-4’-monophosphoryl lipid A (MPL, 50 µg, produced by GSK), *Quillaja saponaria* Molina, fraction 21 (QS-21, 50 µg, Licensed by GSK from Antigenics Inc, a wholly owned subsidiary of Agenus Inc., a Delaware, USA corporation) and CpG 7909 synthetic

oligodeoxynucleotides (ODNs) containing unmethylated CpG motifs (420 µg), in a liposomal formulation. recPRAME (20µg, 100µg or 500µg dose) was co-lyophilized with CpG7909 and reconstituted with the other AS15 components formulated in a 0.5ml solution.

A maximum of 24 doses of PRAME immunotherapeutic could be administered according to the following schedule: the first 6 doses given at 2-week intervals, then 6 doses at 3-week intervals, then 4 doses at 6-week intervals, then every 3 months during year 2 and every 6 months for the next 2 years. Patients will be followed up for safety and clinical outcomes every 3 months for one year, and for survival for 5 years after the first treatment administration (to be published elsewhere).

Enrolment was staggered such that the first 3 patients of each dose-level received their first immunization on different days, allowing earlier identification of safety signals. Escalation to the next dose level occurred when 15 patients had commenced treatment with the previous dose level and when 3 patients had received at least 4 immunizations. Dose escalation only occurred if no case of dose limiting toxicity (DLT) had occurred in the first 3 patients, and if no more than 2 cases of DLT had occurred in all 15 patients enrolled at that dose-level. In the event of one case of DLT in the first 3 patients, the decision to proceed to the next dose-level was postponed until 6 patients at that dose-level had received at least 4 treatment administrations.

Safety monitoring

A Data and Safety Monitoring Committee (DSMC) composed of an independent group of experts reviewed the safety data, the clinical relevance of each DLT event and its relationship to the study treatment. The DSMC made recommendations to the

sponsor concerning the continuation, modification or termination of the trial.

At each visit, blood and urine samples were collected for evaluation of hematologic, biochemical and coagulation parameters including serum cortisol, renal function tests, and urinalysis. Anti-nuclear antibodies were assessed at screening, at the time of doses 6, 12 and 16 and every six months thereafter.

Measurement of humoral immune responses

IgG antibody concentrations found in patient sera were measured in duplicate using a standard ELISA. Briefly, recombinant PRAME produced in *Pichia pastoris* was coated overnight at 2-8°C at 1.5µg/ml in appropriate ELISA plates. After washing and blocking the plates with assay diluent (1% BSA, 0.1% Tween 20, 0.2% Proclin 300 [Supelco]) during 1 hour at room temperature, clinical samples, control and standard samples were diluted in assay diluent complemented with 1mg/ml of *Pichia* lysate. Pre-immunization samples were pre-diluted 1:1000 and post-immunization serum specimens were pre-diluted 1:1000 and 1:10,000. Diluted samples were incubated for 60 minutes (±10 minutes) at room temperature on antigen-coated plates. After washing, a secondary antibody (goat anti-human-IgG antibody conjugated to peroxidase – KPL) was incubated 60 minutes at room temperature. After washing, the chromogen substrate solution (TMB, Biorad) was added and incubated 30 minutes at room temperature. The reaction was stopped with H_2SO_4 (Merck) and absorbance optical density was measured at 450 / 620 nm within 60 minutes. The raw data were analyzed with SoftMaxPro software (Molecular Device). IgG anti-PRAME antibody concentrations were expressed as ELISA Units per milliliter E.U/ml.

Measurement of cell-mediated immune responses

Peripheral blood mononuclear cells (PBMCs) were cultured for 14 days in 24 independent micro-cultures in limiting dilution conditions ($2x10^5$ cells/well), with antigen-specific stimulation (protein immunocomplexed with pool of plasma containing anti-PRAME antibodies) and with IL-2 and IL-7. On day 14, PBMC micro-cultures were divided in two to allow antigen-specific and antigen non-specific (irrelevant) re-stimulation. Antigen-specific stimulation was performed with a pool of 123 15mer peptides (with an overlap of 10 amino acids [aa]) covering the entire PRAME sequence (http://www.uniprot.org/uniprot/P78395), and to which minimal HLA-A2 and HLA-24 CD8 epitopes were added [2, 3]. All peptides were used at 1µg/ml/peptide. Irrelevant re-stimulation was performed with a pool of 43 15mer peptides derived from the NY-ESO-1 protein (http://www.uniprot.org/uniprot/P78358), plus negative control peptides (aa sequences: EGATIVESA and NEGATIVESQNTRQL) to ensure equivalent total peptide mass in both specific and irrelevant stimulation conditions.

CD4+ and CD8+ T-cells in each well were assessed by intracellular flow cytometry for their ability to produce both IFN-γ and TNF-α upon antigen stimulation. Intracellular cytokine staining was performed with the following antibodies: IFN-γ^{FITC}, $CD3^{PercP}$, $TNF^{PE\text{-}cy7}$, $CD4^{APC\text{-}H7}$ and $CD8^{V450}$. Results were analyzed using FlowJo software TreeStar.

The experimental strategy also allowed calculation of approximate frequencies of antigen specific T-cell precursors (assuming a clonal response).

For each pair of wells the ratio between the percentage of double positive cells in the specific and irrelevant stimulation was calculated. The geometric mean of the 24 ratios (GMR, considered as an immunogenicity score) was calculated to integrate

the average responses observed in the 24 independent wells. For wells with fewer than 50 positive antigen-specific events the well ratio was arbitrarily set at 1.

GMR cut-offs were calculated for both CD4+ and CD8+ T cells from 23 healthy donors using the same analysis templates, and values were determined as 2.68 (for CD4+ T cell analysis) and 1.15 (for CD8+ T cell analysis). A patient was considered as an immune responder if the GMR 2 weeks post-dose 4 was both above the cut-off and at least 4 times higher than the GMR at baseline.

Statistical analysis

All statistical analyses were performed using SAS software version 9.2. The study was descriptive and no comparative tests were performed. The total treated cohort included all patients enrolled into the study who had received at least one treatment dose and the according-to-protocol cohort for immunogenicity included all patients who met eligibility criteria, who complied with protocol-defined procedures, and who had received at least the first 4 PRAME immunotherapeutic doses and had completed the visit 2 weeks post-dose 4. Geometric mean antibody concentrations were calculated for anti-PRAME IgG antibodies.

Results

The study commenced on 13 July 2010 and the data lock point for dose selection was 20 December 2011.

References

1. Dyrskjøt L, Zieger K, Kissow Lildal T et al. Expression of MAGE-A3, NY-ESO-1, LAGE-1 and PRAME in urothelial carcinoma. Br. J. Cancer 2012; 107(1):116–122.

2. Ikeda H, Lethé B, Lehmann F et al. Characterization of an antigen that is recognized on a melanoma showing partial HLA loss by CTL expressing an NK inhibitory receptor. Immunity 1997; 6(2):199–208.

3. Kessler JH, Beekman NJ, Bres-Vloemans SA et al. Efficient identification of novel HLA-A(*)0201-presented cytotoxic T lymphocyte epitopes in the widely expressed tumor antigen PRAME by proteasome-mediated digestion analysis. J. Exp. Med. 2001; 193(1):73–88.

Supplementary Figure 1: Patient flow through the study until 2 weeks post-dose 4

Footnote: Missing = no tumor sample received by the laboratory. Inconclusive = invalid test result or quantity not sufficient for testing. SAE = serious adverse event. AE = adverse event. Number who completed the study until 2 weeks after the fourth immunization: 15 in Cohort 1, 14 in Cohort 2 and 21 in Cohort 3.

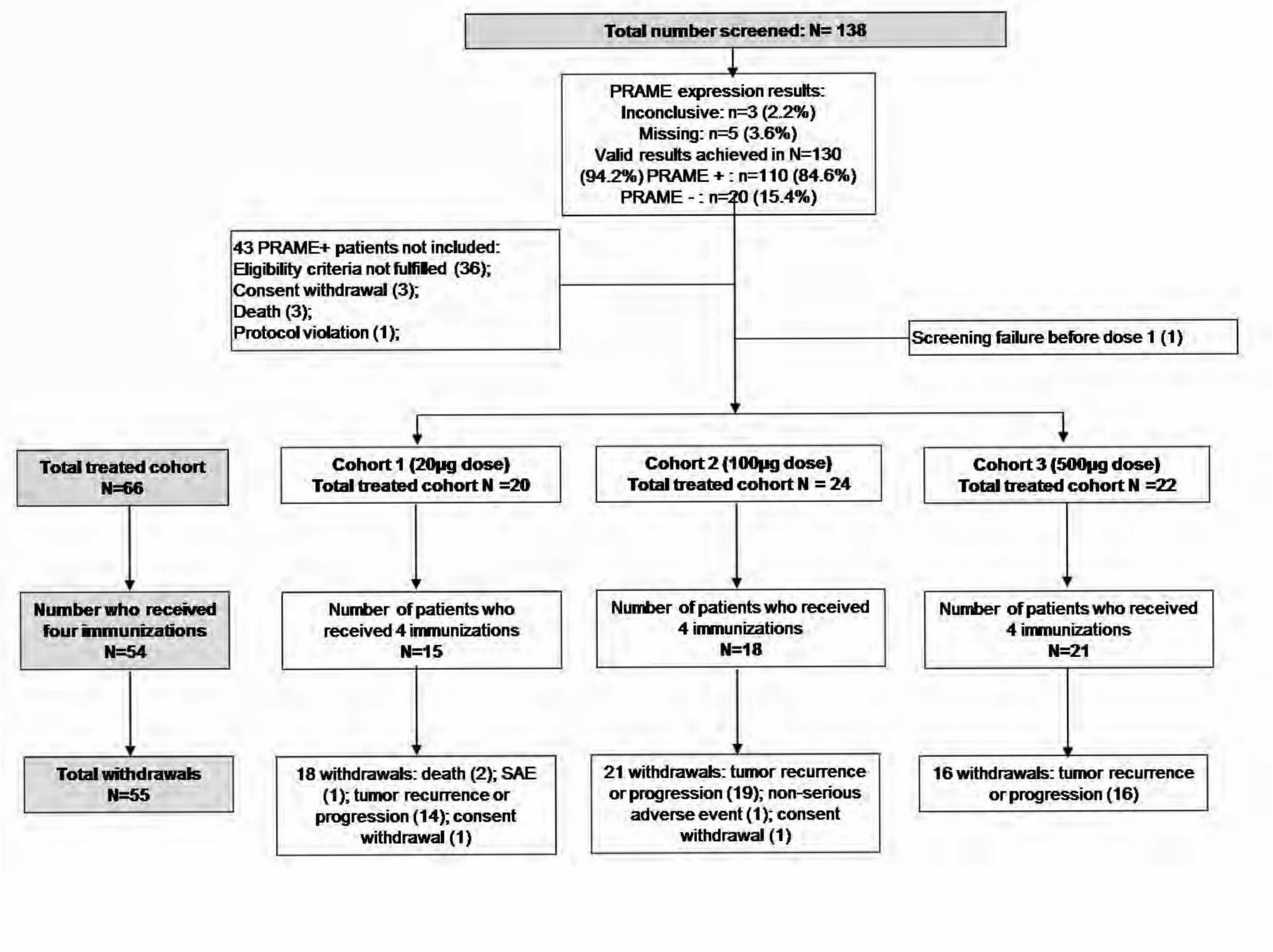

Supplementary Table 1: Number of patients reporting adverse events considered to be treatment related from dose 1 until the data lock point, by maximum grade (Total treated cohort)

	Cohort 1 N = 20			Cohort 2 N = 24			Cohort 3 N = 22		
	Grade 1	Grade 2	Grade 3	Grade 1	Grade 2	Grade 3	Grade 1	Grade 2	Grade 3
**Adverse event**	**n**	**n**	**n**	**n**	**n**	**n**	**n**	**n**	**n**
Not yet coded	1	0	0	1	0	0	1	0	0
Injection site reaction	6	4	0	10	3	0	8	5	0
Pyrexia	3	2	0	5	1	0	5	1	0
Influenza like illness	2	2	0	3	1	0	6	0	0
Fatigue	2	1	0	2	0	0	2	3	0
Headache	4	1	0	0	2	0	1	2	0
Chills	4	0	0	1	0	0	2	2	0
Asthenia	1	0	0	0	0	0	2	4	0
Myalgia	4	0	0	1	1	0	0	1	0
Nausea	5	0	0	1	0	0	0	1	0
Arthralgia	0	1	0	1	1	0	0	0	0
Decreased appetite	1	0	0	1	0	0	1	0	0
Bone pain	1	1	0	0	0	0	0	0	0
Diarrhoea	1	0	0	0	0	0	1	0	0
Oropharyngeal pain	1	0	0	0	1	0	0	0	0
Pain in extremity	0	0	0	0	1	0	0	1	0
Vomiting	1	0	0	0	1	0	0	0	0
Abdominal pain	0	0	0	0	0	0	0	1	0
Abdominal pain upper	0	0	0	0	0	0	1	0	0
Acne	0	1	0	0	0	0	0	0	0
Agitation	0	0	0	1	0	0	0	0	0
Antinuclear antibody increased	0	0	0	1	0	0	0	0	0
Anxiety/agitation	0	1	0	0	0	0	0	0	0
Back pain	0	1	0	0	0	0	0	0	0
Brain oedema	0	0	0	0	0	1	0	0	0
Chest discomfort	1	0	0	0	0	0	0	0	0
Cough	0	0	0	1	0	0	0	0	0
Dissociation	1	0	0	0	0	0	0	0	0
Dizziness	1	0	0	0	0	0	0	0	0
Dry mouth	1	0	0	0	0	0	0	0	0
Dysphagia	0	0	0	1	0	0	0	0	0
Feeling cold	1	0	0	0	0	0	0	0	0
Groin pain	0	1	0	0	0	0	0	0	0
Hyperhidrosis	0	0	0	0	0	0	1	0	0
Hyperthermia	0	0	0	0	0	0	1	0	0
Hypotension	1	0	0	0	0	0	0	0	0
Influenza	1	0	0	0	0	0	0	0	0
Intertrigo	0	0	0	0	0	0	1	0	0
Leukopenia	1	0	0	0	0	0	0	0	0
Lymph node pain	1	0	0	0	0	0	0	0	0
Malaise	0	0	0	1	0	0	0	0	0
Metastatic pain	1	0	0	0	0	0	0	0	0
Microalbuminuria	0	0	0	0	0	0	0	0	1
Musculoskeletal chest pain	0	1	0	0	0	0	0	0	0
Musculoskeletal pain	0	0	0	0	0	0	1	0	0
Neuralgia	0	1	0	0	0	0	0	0	0
Neutropenia	0	1	0	0	0	0	0	0	0

	Cohort 1<br>N = 20			Cohort 2<br>N = 24			Cohort 3<br>N = 22		
	**Grade 1**	**Grade 2**	**Grade 3**	**Grade 1**	**Grade 2**	**Grade 3**	**Grade 1**	**Grade 2**	**Grade 3**
**Adverse event**	**n**	**n**	**n**	**n**	**n**	**n**	**n**	**n**	**n**
Non-cardiac chest pain	1	0	0	0	0	0	0	0	0
Pain	1	0	0	0	0	0	0	0	0
Peripheral coldness	1	0	0	0	0	0	0	0	0
Proteinuria	0	0	0	0	0	0	0	0	1
Pruritus	1	0	0	0	0	0	0	0	0
Rhinitis	1	0	0	0	0	0	0	0	0
Sensation of heaviness	0	0	0	1	0	0	0	0	0
Sensory loss	0	0	0	1	0	0	0	0	0
Tremor	1	0	0	0	0	0	0	0	0
Uterine hemorrhage	1	0	0	0	0	0	0	0	0
Vitiligo	1	0	0	0	0	0	0	0	0
Xerosis	0	0	0	0	0	0	1	0	0

N = number of patients with at least one administered dose

n = number of patients reporting the adverse event at least once
